# Outcome improvement for anaemia and iron deficiency in ERAS hip and knee arthroplasty: a descriptive analysis

**DOI:** 10.1186/s13741-024-00426-3

**Published:** 2024-06-21

**Authors:** Christoffer Calov Jørgensen, Henrik Kehlet, The Center for Fast-track Hip, Knee Replacement Collaborative Group, Torben B. Hansen, Kirill Gromov, Thomas Jakobsen, Claus Varnum, Soren Overgaard, Mikkel Rathsach, Martin Lindberg-Larsen, Manuel Josef Bieder

**Affiliations:** 1 Departement of Anaesthesia and Intensive Care, Hospital of Northern Zeeland, Hillerød, Denmark and The Center for Fast-track Hip and Knee Replacement, Rigshospitalet, Copenhagen, Denmark; 2 Section for Surgical Pathophysiology, Copenhagen, Denmark and The Centre for Fast-Track Hip and Knee Replacement, Rigshospitalet, Copenhagen University, Copenhagen, Denmark

**Keywords:** Anaemia, ERAS, Hip, Knee, Arthroplasty, Outcomes

## Abstract

**Background and purpose:**

Preoperative anaemia including iron deficiency anaemia (IDA) is a well-established perioperative risk factor. However, most studies on iron therapy to treat IDA have been negative and few have been conducted within an enhanced recovery after surgery (ERAS) protocol. Furthermore, patients with IDA often have comorbidities not necessarily influenced by iron, but potentially influencing traditional study endpoints such as length of stay (LOS), morbidity, etc. The aim of this paper is to discuss patient-related challenges when planning outcome studies on the potential benefits of iron therapy in patients with IDA, based upon a large detailed prospective database in ERAS total hip (THA) and knee arthroplasty (TKA).

**Methods:**

A prospective observational cohort study in ERAS THA and TKA from 2022 to 2023. Detailed complete follow-up through questionnaires and electronic medical records.

**Results:**

Of 3655 included patients, 276 (7.6%) had IDA defined as a haemoglobin (Hb) of < 13.0 g/dL and transferrin saturation of 0.20, while 3379 had a Hb of ≥ 13.0. Patients with IDA were a median 5 years older than non-anaemics, with an increased fraction living alone (38.4% vs. 28.8%), using walking aids (54.3% vs 26.4%) and receiving home care (16.2% vs 4.7%). Fewer IDA patients were working (12.7% vs. 29.6%) and a median number of prescribed drugs was higher (10 vs. 6).

Median LOS was 1 day in both IDA and non-anaemic patients, but a LOS of > 2 days occurred in 11.6% of patients with IDA vs. 4.3% in non-anaemics. The proportion with 30- or 90-day readmissions was 6.5% vs. 4.1% and. 13.4% vs6.0%, in patients with IDA and non-anaemics, respectively. However, potentially anaemia or iron deficiency-related causes of LOS > 2 days or 90-day readmissions were only 5.4% and 2.2% in patients with IDA and 1.9% and 1.0% in non-anaemics.

**Conclusion:**

Conventional randomised trials with single or composite “hard” endpoints are at risk of being inconclusive or underpowered due to a considerable burden of other patient-related risk factors and with postoperative complications which may not be modifiable by correction of IDA per se. We will propose to gain further insights from detailed observational and mechanistic studies prior to initiating extensive randomised studies.

## Background

Preoperative anaemia including iron deficiency anaemia (IDA) is a well-established perioperative risk factor, but unfortunately, most studies on iron therapy to IDA in abdominal surgery have been negative (Richards et al. 2023). Similar observations have been made in hip and knee arthroplasty (THA/TKA) (Scrimshire et al. 2020). However, these studies have almost uniformly been done without an Enhanced Recovery After Surgery (ERAS) setting which otherwise is well known to decrease postoperative morbidity (Kehlet 2020). More recently, a well-established implemented ERAS programme with a median length of stay (LOS) of 1 day after THA/TKA in a large series of consecutive patients has demonstrated preoperative anaemia to be a risk factor for postoperative complications using a machine learning setting (Jørgensen et al. 2024).

## Main text

In September 2022, the multicentre fast-track hip and knee replacement collaboration (Jørgensen et al. 2024) initiated a further detailed prospective registration of patient comorbidities including iron status. Here, the preliminary data from the initial 3655 patients having surgery until July 2023 is reported and summarised in Table 1.

**Table 1:** Postoperative outcomes

Postoperative outcomes	Hb ≥ 13 g/dL (*n*: 3379)	IDA (*n*: 276) Hb < 13 g/dL and Tsat < 20%
Median/mean LOS	1/1.0	1/1.4
LOS > 2 days (%)	4.3	11.6
30 days readmission (%)	4.0	6.5
90 days readmission (%)	6.1	13.4
Composite of either LOS > 2 days or 90 days readmissions (%)	10.0	22.8
Patient demographics		
Age (median)	70 (63–76)	75 (68–81)
Working/employed (%)	29.6	12.7
Living alone (%)	28.8	38.4
Home care (%)	4.7	16.2
Use of walking aid (%)	26.4	54.3
> 1 fall < 3 months (%)	5.5	11.1
Pain catastrophizing (median)	15 (7–25)	20 (13–29)
*n* drugs (median)^a^	6 (3–9)	10 (6–13)

Outcome and patient demographics in 3655 patients undergoing total hip and knee arthroplasty

*IDA* iron deficiency anaemia

^a^Number of drugs only validated in 1863 (51.0%) patients at the time of writing

Again, these data within an effective established ERAS setting and complete follow-up clearly demonstrate that patients with IDA have more outcome problems with slightly longer LOS, an increased fraction of patients with LOS > 2 days, 30 days and 90 days readmissions (Table 1). However, the study´s detailed preoperative patient demographic assessments in the lower panel of Table 1 illustrate that in addition to the clear outcome differences, seven other demographics were different in IDA patients vs. the non-anaemic group: a slightly higher age, an increased proportion living alone, an increased proportion receiving home care, fewer having a job, an increased proportion using a walking aid, an increased proportion with two or more falls within 3 months preoperatively and a higher frequency of polypharmacy (> 5 types of drugs).

**Fig. 1:**
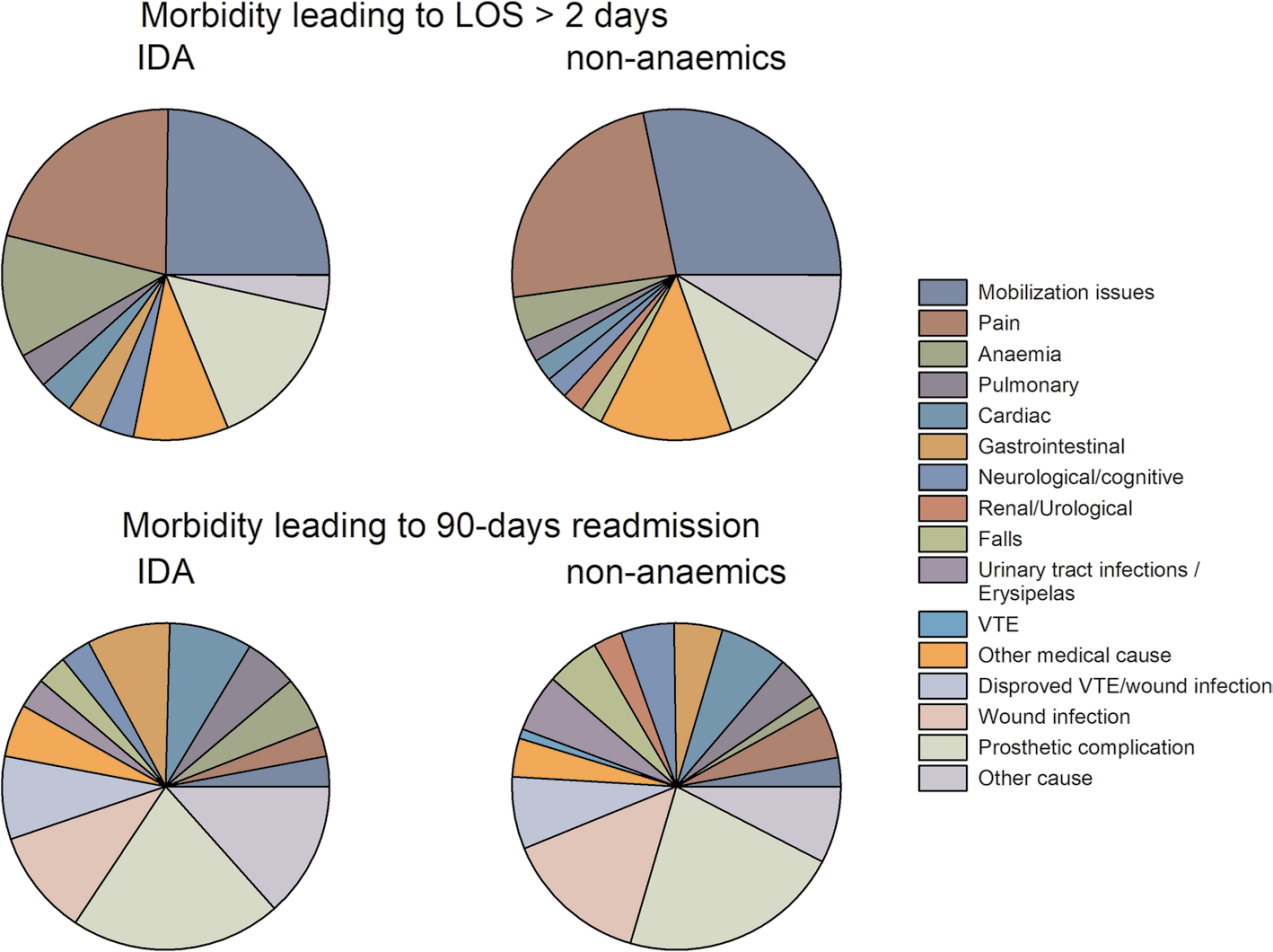
Pie charts of the distributions of causes of LOS > 2 days and 90 days readmissions in IDA and non-anaemic patients

Unfortunately, all of these, except maybe preoperative polypharmacy and fall tendency, maybe non-modifiable within a perioperative setting. Furthermore, the causes of LOS > 2 days and 90 days readmissions were diverse and with a considerable number being related to pain, and surgical complications (Fig. 1). Thus, if planning a conventional all-patient inclusion RCT and with a “hard” composite outcome of LOS > 2 days and 30- to 90-days readmissions, the incidence would be 22.8% according to the presented data. If considering an 8% reduction to be achievable when providing preoperative i.v. iron treatment, 2 × 371 IDA-patients would be needed for a power of 80% and a significance level of 0.05. However, given the considerable amount of postoperative morbidity which may not be related to anaemia or iron deficiency and the additional non-modifiable factors/risk differences without any expected association with the effects of preoperative iron IDA correction, the degree of outcome improvement is dubious.

The two main questions to be answered are therefore how to improve outcomes in IDA patients, and how to measure the potential of iron therapy. Although it is obvious that the fraction of patients presenting with established preoperative IDA should be included in interventional trials, the most suitable trial design and outcome are less straightforward as the conditions for a conventional RCT seem uncertain. This is problematic and may not be easily solved by increasing the number of patients, as increasing the number of recruiting sites or countries may influence outcomes associated with non-protocolised aspects of care (e.g. ± ERAS protocols). Consequently, a classical randomised clinical trial with a composite or single primary “hard” outcome may not be a feasible way forward to study the potential benefits of preoperative iron therapy for patients with IDA. Instead, we may need to consider using hierarchical primary outcomes as seen in some recent studies within internal medicine (Gasparyan et al. 2022), in order to provide information on potential prioritised benefits throughout the peri- and early postoperative period.

Another possibility could be a primary exploratory observational study on complications or improvements with an established/ expected association with anaemia and iron depletion. Such observational studies may serve as a basis before considering a hierarchic or classical RCT. Other potential outcomes of interest include non-invasive VO2_max_ estimation (Sørensen et al. 2020; Plumb et al. 2023) or patient-reported outcome measures (PROMS), despite their limited value compared to the objective assessment of recovery (Wainwright and Kehlet 2022).

## Conclusion

This detailed study in a well-defined ERAS setting demonstrating worse postoperative outcomes in patients with IDA emphasises the need for detailed observational and mechanistic studies on the potential effect of preoperative iron treatment. This is due to the considerable burden of patient-related risk factors which may not be modifiable by correction of IDA alone. Such studies will be important for rethinking study design and outcomes prior to initiating extensive definitive randomised studies (Kehlet and Lobo 2024).

## Data Availability

Data is available in anonymised form from the authors upon request and in accordance with Danish laws and regulations.

## Abbreviations

IDA: Iron deficiency anaemia
THA: Total hip arthroplasty
TKA: Total knee arthroplasty
ERAS: Enhanced Recovery After Surgery
LOS: Length of hospital stay
RCT: Randomised clinical trial
VO2 max: Maximum oxygen consumption rate
PROMs: Patient-reported outcome measures
Tsat: Transferrin saturation
